# Supplementary material for: Resolving the full spectrum of human genome variation using Linked-Reads
Source: Genome Res. 2019 Apr;29(4):635–45. doi: 10.1101/gr.234443.118 (PMC6442396; doi:10.1101/gr.234443.118)
Supplement: Supplemental Material [file supp_gr.234443.118_Supplemental_longranger-2.2.zip › longranger-2.2.tar.bz2!/lr2.2-public/lib/go/src/code.google.com/vitess/docs/resources/roadmap.html]

Vitess / Vitess Roadmap


Toggle navigation

- Overview
- Guides
- Reference
- Blog
- GitHub

- #### Overview

  - What is Vitess
  - Scaling MySQL with Vitess
  - Key Concepts
- #### Getting Started

  - Run Vitess on Kubernetes
    - Custom Docker Build
  - Run Vitess Locally
- #### User Guide

  - Introduction- Client Libraries- Backing Up Data- Reparenting
        - Schema Management
        - VSchema Guide
        - Schema Swap (Tutorial)
        - Sharding
          - Horizontal Sharding (Tutorial, automated)
          - Horizontal Sharding (Tutorial, manual)
          - Sharding in Kubernetes (Tutorial, automated)
          - Sharding in Kubernetes (Tutorial, manual)
        - Vitess Sequences
        - MySQL Server Protocol
        - Vitess and Replication
        - Update Stream
        - Row Based Replication
        - Topology Service
        - Transport Security Model
        - Launching
          - Scalability Philosophy
          - Production Planning
          - Server Configuration
          - 2PC Guide
          - Troubleshooting
        - Upgrading
- #### Reference Guides

  - Vitess API- vtctl Commands
- #### Other Resources

  - Presentations- Blog- Roadmap
- #### Contributing

  - Contributing to Vitess- GitHub Workflow- Code Reviews
- GitHub

# Vitess Roadmap

- #### Overview

  - What is Vitess
  - Scaling MySQL with Vitess
  - Key Concepts
- #### Getting Started

  - Run Vitess on Kubernetes
    - Custom Docker Build
  - Run Vitess Locally
- #### User Guide

  - Introduction- Client Libraries- Backing Up Data- Reparenting
        - Schema Management
        - VSchema Guide
        - Schema Swap (Tutorial)
        - Sharding
          - Horizontal Sharding (Tutorial, automated)
          - Horizontal Sharding (Tutorial, manual)
          - Sharding in Kubernetes (Tutorial, automated)
          - Sharding in Kubernetes (Tutorial, manual)
        - Vitess Sequences
        - MySQL Server Protocol
        - Vitess and Replication
        - Update Stream
        - Row Based Replication
        - Topology Service
        - Transport Security Model
        - Launching
          - Scalability Philosophy
          - Production Planning
          - Server Configuration
          - 2PC Guide
          - Troubleshooting
        - Upgrading
- #### Reference Guides

  - Vitess API- vtctl Commands
- #### Other Resources

  - Presentations- Blog- Roadmap
- #### Contributing

  - Contributing to Vitess- GitHub Workflow- Code Reviews

Vitess is an active open source project. Here is a list of recent and upcoming
features the team is focused on: Check with us on
our forums for more
information.

## Vitess before 2.0 GA

Vitess has been used internally by YouTube for a few years now, but the first
public versions lacked polish and documentation. Version 1.0 was mostly an
internal release.

## Vitess 2.0 GA

Vitess 2.0 GA was
created on July 11, 2016. It was the first major public release.

It contains all the core features of the Vitess product:

- Advanced data access:

  - Automatic query routing to actively serving instances.
  - Scalability through sharding, with transparent access to sharded data,
    without need for proprietary API.
  - Map-Reduce support for data warehousing queries.
  - Limit the negative impact of bad queries.
- Advanced manageability features:

  - Orchestrator integration, auto-reparent on master failure.
  - Out-of-the-box support for Kubernetes
  - No-downtime dynamic resharding.
  - Expose data for advanced monitoring and bad query detection.

## Vitess 2.1

Vitess 2.1 is actively being worked on. Apart from the multiple small changes
we've been making to address a number of small issues, we are adding the
following core features:

- Support for distributed transactions, using 2 phase commit.
- Resharding workflow improvements, to increase manageability of the process.
- Online schema swap, to apply complex schema changes without any downtime.
- New dynamic UI (vtctld), rewritten from scratch in Angular 2. We will still
  have the old UI in Vitess 2.1 as a fallback, but it will be removed in Vitess
  2.2.
- Update Stream functionality, for applications to subscribe to a change stream
  (for cache invalidation, for instance).
- Improved Map-Reduce support, for tables with non-uniform distributions.
- Increase large installation scalability with two-layer vtgate pools (l2vtgate,
  applicable to 100+ shard installations).
- Better Kubernetes support (Helm support, better scripts, ...).
- New implementations of the topology services for Zookeeper (`zk2`) and etcd
  (`etcd2`). In Vitess 2.1 they will become the recommended implementations and
  the old `zookeeper` and `etcd` will be deprecated. In Vitess 2.2 only the new
  `zk2` and `etcd2` implementations will remain, so please migrate after upgrade
  to Vitess 2.1.
- Added support for Consul topology service client.
- Initial version of the Master Buffering feature. It allows for buffering
  master traffic while a failover is in progress, and trade downtime with
  extra latency.

We already cut
the
2.1.0-alpha.1 release,
and we are finalizing the last details at the moment. Release ETA is around
beginning of 2017.

## Vitess Moving Forward

The following list contains areas where we want to focus next, after the current
set of changes. Let us know if one of these areas is of particular interest to
your application!

### Data Access

- Additional support for cross-shard constructs (IN clause, ordering and
  filtering, ...).
- ExecuteBatch improvements (to increase performance of bulk imports for
  instance).
- Multi-value inserts (across shards or not).

### Manageability

- Better support for MySQL DDL constructs.
- Support for MySQL 8.0.
- Additional Kubernetes integration.
- Include resharding workflows in the control panel UI (vtctld).
- Support vertical splits to an existing keyspace, so any table can be moved
  around to any keyspace. Only for unsharded keyspaces, at first.
- Out-of-the-box integration with Promotheus for monitoring.

### Scaling

- Better resource utilization / less CPU usage.

## Future Work

We are considering integration/implementation of the following technologies in
our roadmap. For specific features, reach out to us to discuss opportunities to
collaborate or prioritize some of the work:

- Integration with Mesos and DC/OS.
- Integration with Docker Swarm.
- Improved support for row-based replication (for Update Stream for instance).
- Better integration with Apache Spark (native instead of relying on Hadoop
  InputSource).

Contact: vitess@googlegroups.com  **·**  
Announcements  **·**  
© 2017 Vitess powered by Google Inc
